# A Sensitive LC-MS/MS Method for the Simultaneous Determination of Two Thia-Analogous Indirubin *N*-Glycosides and Indirubin-3′-Monoxime in Plasma and Cell Culture Medium

**DOI:** 10.3390/molecules27093031

**Published:** 2022-05-09

**Authors:** Alica Fischle, Rico Schwarz, Franziska Wendt, Marcel Kordt, Robert Ramer, Lars Boeckmann, Martin Hein, Peter Langer, Steffen Emmert, Brigitte Vollmar, Burkhard Hinz

**Affiliations:** 1 Institute of Pharmacology and Toxicology, Rostock University Medical Center, 18057 Rostock, Germany; alica.fischle@uni-rostock.de (A.F.); rico.schwarz@med.uni-rostock.de (R.S.); franziska.wendt@med.uni-rostock.de (F.W.); robert.ramer@med.uni-rostock.de (R.R.); 2 Rudolf Zenker Institute for Experimental Surgery, Rostock University Medical Center, 18057 Rostock, Germany; marcel.kordt@med.uni-rostock.de (M.K.); brigitte.vollmar@med.uni-rostock.de (B.V.); 3 Clinic and Policlinic for Dermatology, Rostock University Medical Center, 18057 Rostock, Germany; lars.boeckmann@med.uni-rostock.de (L.B.); steffen.emmert@med.uni-rostock.de (S.E.); 4 Institute of Organic Chemistry, University of Rostock, 18059 Rostock, Germany; martin.hein@uni-rostock.de (M.H.); peter.langer@uni-rostock.de (P.L.)

**Keywords:** thia-analogous indirubin *N*-glycosides, validation, protein precipitation, liquid-liquid extraction, LC-MS/MS

## Abstract

Indirubin was identified as an active component of Danggui Longhui Wan, an herbal mixture used in traditional Chinese medicine, and showed anticancer activity in clinical trials in patients with chronic leukemia. Investigations on the mechanisms of antitumor action of indirubins have mainly focused on the indirubin derivative indirubin-3′-monoxime (I3M). Meanwhile, antiproliferative and cytotoxic properties on cancer cells have also been demonstrated for several synthetic indirubin *N*-glycosides. In the present study, we demonstrate cytotoxic activity of the thia-analogous indirubin *N*-glycosides KD87 (3-[3′-oxo-benzo[*b*]thiophen-2′-(*Z*)-ylidene]-1-(β-d-glucopyranosyl)-oxindole) and KD85 (3-[3′-oxo-benzo[*b*]thiophen-2′-(*Z*)-ylidene]-1-(β-d-mannopyranosyl)-oxindole) against melanoma and squamous cell carcinoma cells as well as lung cancer and glioblastoma cells. The advanced state of preclinical studies on the effects of indirubins conducted to date underscores the need for pharmacokinetic data from cellular, animal, and human studies for which reliable quantification is required. Therefore, a sensitive liquid chromatography-tandem mass spectrometric (LC-MS/MS) method was developed and validated for the simultaneous measurement of KD87, KD85, and I3M in plasma and cell culture medium. Experimental conditions for sample preparation were optimized for human plasma protein precipitation and liquid-liquid extraction from plasma and cell culture medium. The methods were successfully validated in accordance with the U.S. Food and Drug Administration Bioanalytical Method Validation and evaluated for selectivity, sensitivity, matrix effect, recovery, carryover, calibration curve linearity, accuracy, precision, and stability. The applicability of the methods was demonstrated by the determination of KD87 in mouse plasma after prior intraperitoneal administration to mice.

## 1. Introduction

**Figure 1:**
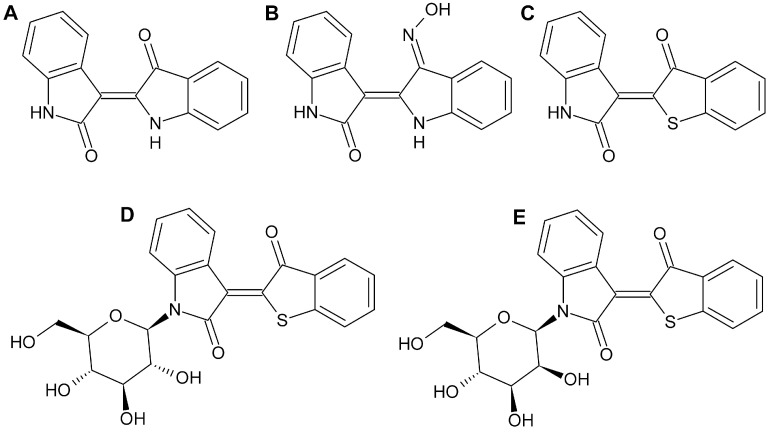
Chemical structures of indirubin ((**A**), internal standard), indirubin-3′-monoxime ((**B**), I3M), KD88 ((**C**), 3-[3′-oxo-benzo[*b*]thiophene-2′-(*Z*)-ylidene]-oxindole; non-glycosylated indirubin thia-analogue), KD87 ((**D**), 3-[3′-oxo-benzo[*b*]thiophen-2′-(*Z*)-ylidene]-1-(β-d-glucopyranosyl)-oxindole; thia-analogous indirubin *N*-glycoside with glucose as sugar component), and KD85 ((**E**), 3-[3′-oxo-benzo[*b*]thiophene-2′-(*Z*)-ylidene]-1-(β-d-mannopyranosyl)-oxindole; thia-analogous indirubin *N*-glycoside with mannose as sugar component).

Indirubin, a 3,2′-bisindole isomer of 2,2′-bisindole indigo (Figure 1), is an active component of Danggui Longhui Wan, an herbal mixture used in traditional Chinese medicine, and has shown significant anticancer activity in chronic myeloid and chronic granulocytic leukemia in clinical trials (for review see [1,2]). Meanwhile, for the cell-permeable indirubin-3′-monoxime (I3M, Figure 1) or its derivatives, a number of intracellular proteins such as cyclin-dependent kinases [3], glycogen synthase kinase 3 [4], or signal transducer and activator of transcription-3 [5,6] have been identified as possible targets of antitumor activity. Based on the isolation of *N*-glycosides of indirubin from terrestrial *Streptomyces* sp. GW 48/1497 [7], a series of indirubin *N*-glycosides [8], thia-analogous indirubin *N*-glycosides [9,10], and *N*-glycosylated 3-alkylidene oxindoles [11,12,13,14] were synthesized, which were found to be antiproliferative and cytotoxic against various tumor cell lines. Thereby, indirubin *N*-glycosides showed higher antiproliferative activity on MCF-7 breast cancer cells [8] than non-glycosylated indirubin [15], with different assays and incubation times used in these studies. Moreover, detailed pathways of apoptosis induction in melanoma cells have meanwhile been described for *N*-glycosylated 3-alkylidene oxindoles [10,13]. The potential of indirubin derivatives for skin cancer therapy was recently summarized in a comprehensive review [16].

The advanced preclinical cellular studies on indirubins highlight the need for perspective kinetic studies on indirubin derivatives in animals and possibly later in humans, requiring the development and validation of appropriate analytical methods to detect drug concentrations in plasma. In addition, such studies could provide valuable information on the kinetics of indirubins in cells and, in conjunction with appropriate extraction procedures for cellular components, on their intracellular distribution. In the present study, a liquid chromatography-tandem mass spectrometric (LC-MS/MS) method was developed for the simultaneous measurement of the indirubin *N*-glycosides KD87 (3-[3′-oxo-benzo[*b*]thiophen-2′-(*Z*)-ylidene]-1-(β-d-glucopyranosyl)-oxindole; Figure 1) and KD85 (3-[3′-oxo-benzo[*b*]thiophene-2′-(*Z*)-ylidene]-1-(β-d-mannopyranosyl)-oxindole; Figure 1) as well as I3M (indirubin-3′-monoxime; Figure 1) in plasma and cell culture medium. Indirubin (Figure 1) served as an internal standard. Validation was performed in accordance with U.S. Food and Drug Administration (FDA) guidance [17].

## 2. Results and Discussion

### 2.1. Effect of Thia-Analogous Indirubin N-Glycosides on the Viability of Various Tumor Cells

**Figure 2:**
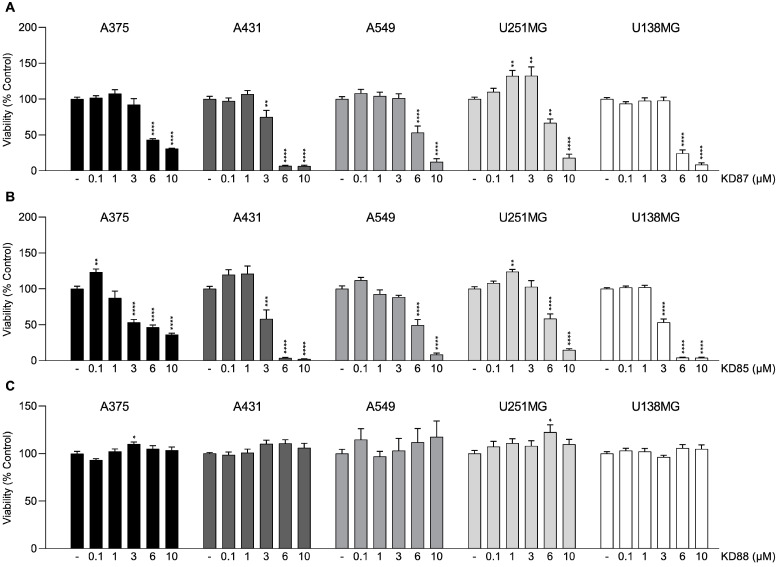
Effect of increasing concentrations of KD87 (**A**), KD85 (**B**), and KD88 (**C**) on viability of various cancer cells. A375 (melanoma; black columns), A431 (skin squamous cell carcinoma; dark grey columns), A549 (non-small cell lung carcinoma; grey columns), U251MG (glioblastoma; light grey columns), or U138MG (glioblastoma; white columns) were incubated for 48 h with the appropriate compounds or vehicle followed by determination of cell viability by WST-1 assay. Percent control represents comparison with vehicle-treated cells (100%) in the absence of test substance. Values are mean ± standard error of the mean (SEM) of *n* = 15 in all panels, except *n* = 14 for A375 and A431 cells in panel B and *n* = 14–15 for A375 cells in panel C, from four (A375, A431) or five (A549, U251MG, U138MG) independent experiments. * *p* ≤ 0.05, ** *p* ≤ 0.01, *** *p* ≤ 0.001, **** *p* ≤ 0.0001 vs. vehicle control; one-way ANOVA with Dunnett’s post hoc test.

To test the biological activity of the thia-analogous indirubin *N*-glycosides that are the focus of the present work, the effects of KD87 and KD85 and their non-glycosylated parent compound KD88 on the viability of various tumor cell lines were initially investigated. The corresponding analyses were performed using the cell lines A375 (melanoma), A431 (skin squamous cell carcinoma), A549 (non-small cell lung carcinoma), U251MG (glioblastoma), and U138MG (glioblastoma). The WST-1 colorimetric assay, performed after 48-h incubation of tumor cells with the compounds, detects the cleavage of WST-1, a water-soluble tetrazolium salt (4-[3-(4-iodophenyl)-2-(4-nitrophenyl)-2H-5-tetrazolio]-1,3-benzenedisulfonate), to a soluble formazan dye by metabolically active cells and can therefore be considered a measure of cell viability. As shown in Figure 2, both KD87 and KD85 showed significant cytotoxic effects starting at a concentration of 6 µM in all cell lines tested and even at 3 µM in some cell lines, particularly in the case of KD85. On the other hand, no cytotoxic potential could be found for the non-glycosylated indirubin thia-analogue KD88.

These data show good agreement with the cytotoxic properties of thia-analogous indirubin *N*-glycosides including KD87 and KD85 registered in previous work using the melanoma cell lines SK-Mel-19, SK-Mel-29, SK-Mel-103, and SK-Mel-147 [9], and extend the spectrum of activity of these compounds to other tumor cell lines such as squamous cell carcinoma, non-small cell lung carcinoma, and glioblastoma. There is also agreement in the literature with regard to the lack of tumor cell toxic effect of the non-glycosylated thia-analogous indirubin KD88. Accordingly, in a previous study using the sulforhodamine B cytotoxicity assay [15], IC_50_ values for unsubstituted indirubin (Figure 1) were 31 µM (A549 lung cancer cells), 42 µM (human fibrosarcoma cell line HT1080), and >100 µM for human gastric (SNU-638), colon (Col2), and breast cancer cells (MCF-7), as well as leukemia cells (HL60). Remarkably, in the cited publication, the incubation period was even 24 h longer than in the assay we used.

### 2.2. Method Development

In the literature, protein precipitation is not as common for the release of indirubin derivatives and analogs from plasma, and it has even been reported to be less efficient than liquid-liquid extraction [18]. However, in a recently published approach to develop an analytical method for measuring 6-bromoindirubin-3′-oxime, plasma samples were pretreated by protein precipitation with a mixture of acetonitrile:methanol (9/1) [19]. In view of these divergent reports, protein precipitation was investigated as an alternative approach for rapid preparation and quantification of analytes of interest in human plasma.

On the other hand, there are a number of methods developed for the analytical characterization of indirubin derivatives and analogues using the principle of liquid-liquid extraction for sample preparation [18,20,21,22]. In this regard, previous publications reported the successful extraction of indirubin derivatives from rat plasma using ethyl acetate [18,22], while the extraction of 5-methylindirubin from mouse plasma was performed using diethyl ether [20]. Park et al. [21] described the extraction of 5-nitro-5′-hydroxy-indirubin-3′-oxime from human plasma using methyl tert-butyl ether as a solvent. Another approach was a combination of both methods for the preparation of rat plasma, as reported by Kim et al. [23], who performed liquid-liquid extraction with ether/dichloromethane followed by protein precipitation of human plasma samples with acetonitrile. To our knowledge, however, there are no known procedures for the extraction of glycosylated indirubin analogues from plasma and cell culture medium. Therefore, and considering the biological activity demonstrated for these compounds, the aim of the present work was to develop a method for liquid-liquid extraction of the corresponding compounds from human plasma as well as from cell culture medium, where we were interested in Dulbecco’s Modified Eagle Medium (DMEM) as a particularly common medium.

The corresponding processing methods established by us were performed with the three analytes I3M, KD87, and KD85, respectively. Due to its structural similarity, indirubin was chosen as the internal standard for all sampling methods. For protein precipitation, the optimal precipitant and the appropriate duration of the shaking and centrifugation steps were investigated. In addition, an acidification step was included in the procedure. Liquid-liquid extraction was adjusted for the suitability of the extraction agent, the possible influence of pH, the duration of the shaking and centrifugation steps, the optimal evaporation conditions, and the composition of the reconstitution solvent. The LC conditions considered column type suitability, gradient elution program, autosampler and oven temperature, injection volume, and post-injection needle purge volume. The MS parameters were optimized by preparing a substance standard in methanol that was continuously injected during method optimization. Measurement was performed with both positive and negative electrospray ionization to investigate the appropriate sensitivity of ionizability of each compound. For KD87, KD85, and indirubin, [M + H]^+^ transitions were found to be appropriate for multiple reaction monitoring (MRM) transitions, whereas I3M was monitored for [M + H]^+^ transitions in plasma samples and [M − H]^−^ transitions upon extraction from DMEM, where more consistent sample signals were observed. LC and MS parameters with observed retention times and quantified ion transitions are listed in Table 1.

### 2.3. Calibration Curve

In all calibrations, quantification was performed by determining the peak area ratios of the MS/MS fragmentations of the internal standard and the quantifiers of each analyte. The calibration curve was generated by a simple linear regression which included a 1/x weighting using the least squares method. The coefficient of determination was at least 0.99 or better in all validation runs.

For precipitated plasma samples, the concentration of analytes ranged from 0.67 to 333 nM, whereas for extracted plasma samples, the linearity of KD87, KD85, and I3M ranged from 1 to 500 nM. The concentration of analytes extracted from DMEM ranged from 1 to 50 nM and contained twelve levels including the zero calibrator.

### 2.4. Selectivity and Carryover

To prove selectivity, blanks from six different human plasmas were processed by protein precipitation or liquid-liquid extraction. In addition, two different DMEM media with high glucose content were measured to demonstrate the selectivity of DMEM extraction. No MRM transitions were detected for the individual retention times of the substances. This confirms the selectivity of the method. 

**Figure 3:**
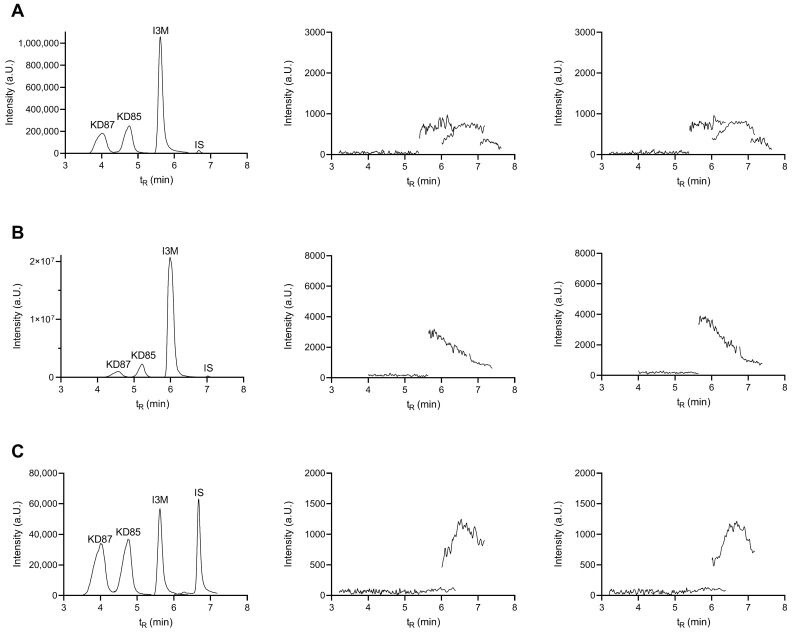
Representative total ion chromatograms (TIC) for (**A**) carryover after plasma precipitation at 333 nM, (**B**) carryover after extraction from plasma at 500 nM, and (**C**) carryover after extraction from DMEM at 50 nM. The indirubin concentration was always 50 nM. Shown on the left are the TIC for all analytes (elution order listed in Table 1). Shown in the middle is the TIC of a blank run with acetonitrile/water (50/50) after one injection of sample. Shown on the right is the TIC of a blank run with acetonitrile/water (50/50) after double injection of the sample. The injection volume was always 10 µL. TIC are shown in ESI+ except for DMEM extracted I3M (ESI−).

As shown in the total ion chromatograms presented in Figure 3, no carryover of analytes and internal standard from one LC-MS/MS run to the next was observed throughout the calibration curve.

### 2.5. Sensitivity, Accuracy and Precision

**Figure 4:**
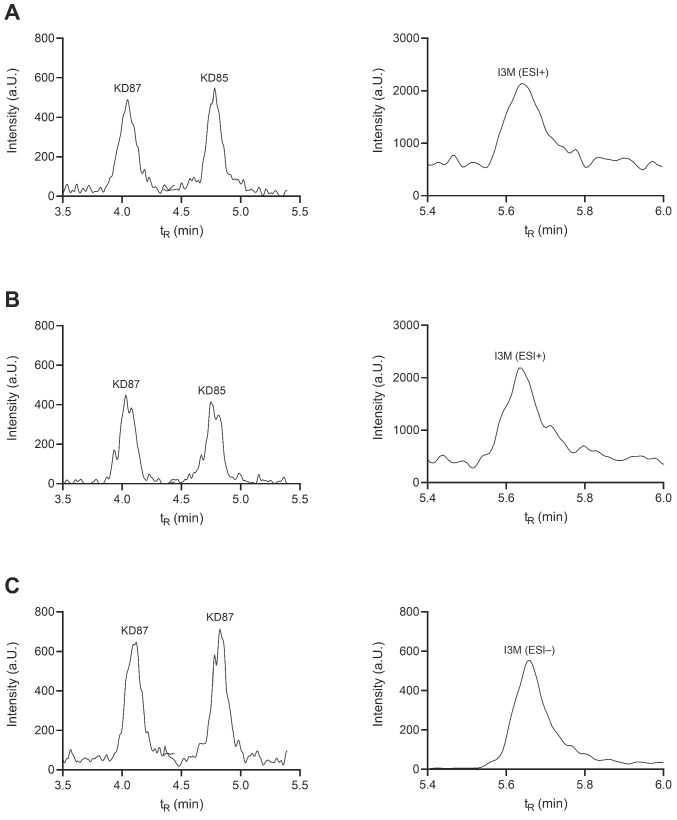
Representative total ion chromatograms for LLOQ after acidic plasma precipitation (**A**), alkaline plasma extraction (**B**), and alkaline DMEM extraction (**C**). On the left side, the peaks of KD87 and KD85 at 0.67 nM (0.295 ng/mL) (**A**), 1 nM (0.441 ng/mL) (**B**), and 1 nM (0.441 ng/mL) (**C**), respectively, are shown. On the right side, the signal is shown for I3M (ESI+) at 0.67 nM (0.152 ng/mL) (**A**,**B**) and for I3M (ESI−) at 1 nM (0.277 ng/mL) (**C**), respectively.

The LLOQ of precipitated plasma was lowest; here, KD87, KD85, and I3M had an LLOQ of 0.67 nM. For the analytes extracted from plasma and DMEM, the LLOQ for all analytes was determined to be 1 nM. The signal-to-noise ratio (S/N) of the LLOQ was at least 5 in each validation run, as required by FDA guidance [17]. S/N values were determined by dividing the difference between analyte signal and background noise with the square root of the background noise. According to Figure 4, the sensitivity requirements were met for each of the three processing methods.

The lowest S/N was observed for the monitored ion transfers after precipitation of plasma samples, with KD87 having a S/N of 17.6, KD85 a S/N of 25.9, and I3M+ a S/N of 9.0. In contrast, the S/Ns for analytes extracted from plasma at 1 nM were 31.2 (KD87), 50.1 (KD85), and 14.5 (I3M+). Finally, for DMEM extraction, all analytes had an LLOQ at 1 nM. Here, KD87 presented a S/N of 16.9, KD85 a S/N of 24.5, and I3M in negative ESI a S/N of 162.8. The sensitivities obtained were comparable to previously reported method performances for both protein precipitation [23] and liquid-liquid extraction [18,20,22].

The inter- and intra-day accuracy and precision for the LLOQ, low, medium, and high concentration for each analyte and method are shown in Table 2, Table 3 and Table 4. Since each sample processing method has a specific calibration range, the control concentration levels were matched specifically to the requirements of the respective procedure. For each intra-day, a total of six replicate samples were prepared per control concentration, of which at least five were included in assessment of accuracy and precision. All values from four intra-day evaluations were combined to assess inter-day accuracy and precision of the method. The FDA requirements that relative errors (RE) should not exceed ±15% in each validation run, with the exception of LLOQ at ±20% [17], were always met. Additionally, the coefficients of variation (CV) should not deviate more than 15% according to the FDA guideline [17], except for the LLOQ of 20%. This criterion was also met for all four concentration levels for both intra-day and inter-day specifications (Table 2, Table 3 and Table 4).

### 2.6. Recovery and Matrix Effect

For recovery determination, the areas of samples spiked with the analytes and subsequently processed were compared with those of processed matrix samples spiked with the analyte only after processing. The highest recoveries were obtained after precipitation of plasma samples, while extraction of the analytes from plasma resulted in slightly lower recoveries. The lowest analyte recoveries were determined for extracted DMEM samples.

Similar high recoveries were observed for KD87, KD85, and I3M at all three concentration levels when precipitating plasma samples. For KD87, recoveries were between 86.64% and 97.77%. KD85 was recovered between 87.67% and 90.20%. For I3M, recoveries were always above 92.34%. At 50 nM, indirubin was recovered with 97.0%.

In comparison, analytes extracted from plasma at 3 nM showed comparable recoveries for all analytes throughout the control concentration levels. In contrast to plasma precipitation, indirubin was recovered from extracted plasma with 64.3% at 50 nM.

For analytes extracted from DMEM, recovery was in general lower for all analytes compared to plasma precipitation and plasma extraction, while indirubin was recovered at 83.4% when 5 µL was injected and at 85.4% when 2 µL of sample was injected.

The corresponding recovery values are summarized in Table 5.

The matrix effect was analyzed using three concentration levels of analytes and the internal standard in three to five different samples. To this end, the absolute values of the areas of extracted matrix samples, which were subsequently spiked with the respective analyte, were compared to the areas of non-extracted analyte standards. The observed ionization suppression was minimal for all analytes and each of the three sample processing methods. 

For KD87 extracted from plasma, the matrix effect was small at 0.94% (3 nM), −0.08% (40 nM), and 3.15% (250 nM). For DMEM extraction, the matrix effect of KD87 was 0.68% (3 nM), 1.20% (15 nM), and 3.95% (40 nM). A similar low matrix effect was observed for KD85 extracted from plasma, which was 0.61% (3 nM), 1.01% (40 nM), and 1.31% (250 nM). Extraction of KD85 from DMEM resulted in a matrix effect of −0.61% (3 nM), 5.97% (15 nM), and 1.74% (40 nM). For extraction from plasma, the matrix effect on I3M was −0.55% at 3 nM, 2.57% at 40 nM, and −1.12% at 250 nM. For I3M in DMEM, the observed matrix effect was similarly low at 1.46% (3 nM), 2.93% (15 nM), and 1.83% (40 nM). For the internal standard indirubin, the matrix effect was determined at 50 nM for extraction from plasma and DMEM, reaching 2.68% for extraction from plasma and approximately 2% at 5 and 2 µL injection volumes for extraction from DMEM.

The corresponding values for the matrix effect are summarized in Table 6.

### 2.7. Stability

To assess stability, low and high concentrations of all analytes were subjected to different storage conditions. The analytes were considered stable if the nominal concentration did not variate more than ±15%. For determination of long-term stability, samples were stored in either plasma or DMEM at –80 °C for 90 and 91 days, respectively. To measure short-term stability, samples were stored at room temperature, in an autosampler, at 4 °C, and at –20 °C for 24 h. In addition, freeze-thaw cycles were performed according to FDA guidance [17] for the standard solutions. For comparison, at least three freshly prepared samples were prepared for each concentration level. With the exception of autosampler stability, the internal standard was always added to all samples before sample processing was started.

Based on the storage investigations listed in Table 7, plasma samples should not be stored above 0 °C to ensure analyte stability over time. In comparison, the analytes in DMEM were more sensitive to storage at lower temperature conditions during short-term storage. With regards to long-term storage at –80 °C, all analytes were stable regardless of matrix. Since autosampler stability after liquid-liquid extraction of samples from both plasma and DMEM can be considered inconsistent, immediate analysis of samples after completion of the preparation procedure is appropriate. Contrary to this observation, both thia-analogous *N*-glycosides were stable in the autosampler after precipitation of plasma samples. Thus, stability in the autosampler appears to be specific to this preparation procedure. 

### 2.8. Quantification of KD87 in Plasma from Mice Treated with KD87 Intraperitoneally

To apply the method validated for plasma, an animal experiment was performed in which KD87 (20 mg/kg body weight) was injected intraperitoneally into NOD.Cg-*Prkdc^scid^ Il2rg^tm1Wjl^*/SzJ (NSG) mice followed by determination of the plasma concentration of KD87 at two fixed time points, i.e., 30 and 60 min, after administration. For this initial investigation, KD87 was selected because it has shown viability-reducing effects on a large panel of tumor cells both in the present study and in a previous study [9], making it a good candidate for future in vivo experiments. 

**Figure 5:**
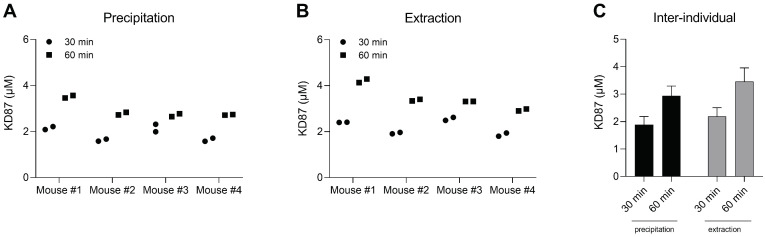
Quantification of KD87 in plasma of mice 30 and 60 min after intraperitoneal injection of KD87 (20 mg/kg body weight). Prior to sample processing, mouse plasma was diluted 1:5 with human plasma. Shown are the individual values of KD87 in mouse plasma (**A**) after protein precipitation and (**B**) after liquid-liquid extraction, as well as (**C**) the average KD87 concentration values of all four mice for both processing methods. (**A**,**B**): The values of two separately processed plasma aliquots of one blood sample per mouse are shown. (**C**): The bar chart summarizes the results of measurements from panels (**A**,**B**), expressed as mean ± standard deviation (SD) of *n* = 8 values per column.

Before starting plasma extraction and protein precipitation, 10 µL of the sample plasma was mixed with 40 µL of human plasma. After appropriate sample preparation by protein precipitation or liquid-liquid extraction, KD87 was successfully detected and quantified by LC-MS/MS. Here, for each blood draw, two separate plasma aliquots were extracted or precipitated and subsequently measured. In addition, the MRM transitions of the aglycone KD88 were monitored during the measurement to observe possible deglycosylation of KD87. No signals of KD88 were detected. Individual concentrations are presented for each mouse separately (Figure 5A,B) and as averages of the four treated mice (Figure 5C). For protein precipitation, a concentration of 1.89 ± 0.30 µM was determined 30 min after intraperitoneal injection, increasing to 2.93 ± 0.36 µM after 60 min (Figure 5C). Similar values are obtained after liquid-liquid extraction of plasma sample, which yielded a concentration of 2.19 ± 0.32 µM and 3.46 ± 0.50 µM after 30 and 60 min, respectively (Figure 5C).

As evident from the average values of all mice (Figure 5C), the KD87 concentration is slightly higher after plasma extraction. This could be due to the presence of ethanol during treatment application, possibly facilitating the transition of the drug to the organic phase during extraction. This effect is negligible during plasma precipitation, as KD87 remains in solution and does not transfer to another solvent. However, considering the inter-individual variations, both quantification methods provide reliable results.

## 3. Materials and Methods

### 3.1. Chemical Reagents

All three thia-analogous indirubins were synthesized by the group of Prof. Peter Langer (Institute of Organic Chemistry, University of Rostock, Rostock, Germany). Indirubin and indirubin-3′-monoxime with a purity of ≥98% were purchased from Cayman Chemical Company (Hamburg, Germany). Acetonitrile was from J. T. Baker (Gliwice, Poland). Water and ethyl acetate were bought from Merck (Darmstadt, Germany). Formic acid was obtained from Honeywell Fluka (Seelze, Germany). All reagents used for HPLC separation were of ultragradient HPLC grade. A 0.5 M acidic buffer consisting of H_2_SO_4_ saturated with Na_2_SO_4_ and a 0.8 M alkaline carbonate buffer consisting of NaHCO_3_ and Na_2_CO_3_ were freshly prepared. High-glucose Gibco™ DMEM (4.5 g/L glucose, GlutaMAX™-I supplement and pyruvate; #31966021) was purchased from ThermoFisher Scientific, Inc. (Schwerdte, Germany) and high-glucose BioWhittaker^TM^ DMEM (4.5 g/L glucose, UltraGlutamine^TM^ I and pyruvate; #BE12-604F/U1) was purchased from Lonza Cologne GmbH (Cologne, Germany). Fetal bovine serum (FBS) superior was purchased from Bio&Sell GmbH (Feucht, Germany) and penicillin-streptomycin was obtained from Sigma-Aldrich Corporation (Taufkirchen, Germany). Dulbecco’s phosphate-buffered saline (DPBS) was obtained from PAN-Biotech GmbH (Aidenbach, Germany). Human plasma was provided by the Institute of Transfusion Medicine at the Rostock University Medical Center.

### 3.2. Cell Culture and Viability Assay

The human melanoma cell line A375 (#300110; RRID:CVCL_0132) as well as the human skin epidermoid carcinoma cell line A431 (#300112; RRID:CVCL_0037) were purchased from CLS Cell Lines Service GmbH (Eppelheim, Germany). The human glioblastoma cell line U251MG (#09063001; RRID:CVCL_0021) was from Sigma-Aldrich Corporation. The human glioblastoma cell line U138MG (#ACC 291; RRID: CVCL_0020) and the human lung carcinoma cell line A549 (#ACC 107, RRID:CVCL_0023) were purchased from DSMZ (Deutsche Sammlung von Mikroorganismen und Zellkulturen GmbH, Braunschweig, Germany).

Cells were cultured in high-glucose DMEM (with GlutaMAX™-I: A375, A431; with UltraGlutamine^TM^ I: A549, U251MG, U138MG) supplemented with 10% heat-inactivated FBS, 100 U/mL penicillin, and 100 μg/mL streptomycin and grown in a humidified incubator at 37 °C and 5% CO_2_. For viability analyses, cells were seeded in 96-well plates at a density of 5000 cells per well in DMEM containing 10% heat-inactivated FBS and cultured for 24 h. All cell experiments with the specific substances were performed in serum-free DMEM supplemented with 100 U/mL penicillin and 100 μg/mL streptomycin, applied after washing the cells with DPBS. The test substances were dissolved in DMSO. The final concentration of DMSO in the incubation media of test substance- and vehicle-treated cells was 1% (*v*/*v*). 

After completion of the 48-h incubation, WST-1 reagent (Sigma, Taufkirchen, Germany) was added to the cells to achieve a final dilution of 1:10, and the cells were incubated for an additional 20 min before absorbance was measured at 450 nM, reference wavelength 690 nM, using a microplate reader.

### 3.3. Standard Preparation

Stock solutions of KD87, KD85, and I3M were prepared in a range from 0.4 µM to 200 µM by serial dilution with acetonitrile (0.4, 0.8, 1.2, 2.0, 4.0, 6.0, 8.0, 10, 12, 16, 20, 40, 70, 100, and 200 µM). Working solutions were prepared by diluting each stock solution 1:100 in human plasma or DMEM. 

Regarding the medium, validation procedures were performed with high-glucose DMEM supplemented with 100 U/mL penicillin and 100 μg/mL streptomycin, but not with FBS. With the exception of selectivity, where both media (GlutaMAX™-I or UltraGlutamine^TM^ I supplement) were tested, only Gibco™ DMEM with GlutaMAX™-I was used for all other analytical procedures.

The internal standard was prepared for both preparation methods by diluting a 10 mM stock solution to 100 µM. For extraction, a 1 µM working solution was prepared, while a 1.5 µM standard solution was used for precipitation. After sample processing, the final concentration of the internal standard was 50 nM. All preparative dilution steps were performed in acetonitrile. Sample processing started with the addition of 10 µL indirubin standard to 50 µL analyte working solution.

After mixing the 1.5 µM internal standard and the plasma sample, protein precipitation was commenced. Next, 190 µL of acetonitrile and 50 µL of 0.5 M sulfuric acid buffer were added. The tubes were then shaken at 1400 rpm for 5 min followed by centrifugation at 14,000 rpm for 5 min. Subsequently, 195 µL of the supernatant was transferred to an LC tube and analyzed. 

Liquid-liquid extraction was performed identically for plasma and DMEM samples. After addition of the 1.0 µM internal standard, 50 µL of 0.8 M alkaline carbonate buffer was added. The extraction was performed once with 890 µL ethyl acetate. Tubes were then shaken for 10 min at 1400 rpm using a Thermomixer 5436 (Eppendorf AG, Hamburg, Germany) followed by centrifugation for 5 min at 14,000 rpm using a Centrifuge 5415 C (Eppendorf AG, Hamburg, Germany). Subsequently, 850 µL of the supernatant was transferred to a new tube and evaporated to dryness for 35 min at 35 °C (8 min heating time) using a SpeedVac SPD130DLX (Thermo Fisher Scientific, Asheville, NC, USA). Reconstitution was performed with 200 µL of a acetonitrile/water/H_2_SO_4_ [3/0.5/0.5] mixture, shaken at 1400 rpm for 5 min, and centrifuged again at 14,000 rpm for 5 min. Finally, 155 µL of the sample was transferred to an LC vial and analyzed.

### 3.4. LC-MS/MS Analysis

The analysis was performed using a Prominence LC-20AD system (Shimadzu, Duisburg, Germany). Separation was carried out on a Multospher^®^ 120 RP 18 AQ-5µ (125 × 2 mm, 5 µm particle size) equipped with a guard column consisting of a Multospher^®^ 120 RP 18 AQ-5µ cartridge (20 × 2 mm, 5 μm particle size), both purchased from CS-Chromatographie Service GmbH, Langerwehe, Germany. Solvent A was water and solvent B was acetonitrile, both containing 0.2% formic acid. The validated elution gradient was run at a flow rate of 0.5 mL/min. It started at 30% B, increased to 35% B for 2.5 min, was driven to 45% B within one min, then increased to 60% B for 1.5 min, reached 100% B in the seventh min, and dropped to 30% B within 0.01 min. The re-equilibration time was 2.99 min. One run was completed in 10 min. The autosampler temperature was 23.0 °C, while the oven temperature was 60.0 °C. For the measurement of the extracted and precipitated plasma samples, 5 µL were injected, and for the analytes extracted from DMEM, an additional run with 2 µL injection volume was performed for validation of I3M. Since the detector’s upper limit for the higher injection volume was exceeded, this parameter was lowered to 2 µL. The HPLC system was connected to an LCMS-8050 triple quadrupole mass spectrometer (Shimadzu, Duisburg, Germany). KD87 and KD85 were ionized by ESI in positive mode, and I3M was additionally ionized in negative mode. Multiple reaction monitoring (MRM) was used to identify and quantify the analytes. The substances were introduced to the mass spectrometer with a nebulization gas flow of 3.0 L/min. The interface voltage was 4.0 kV, and the interface temperature was 300 °C. The heating and drying gas flow was 10.0 L/min, and desolvation was performed at 526 °C. The analysis was carried out using LabSolutions LCMS analysis software from Shimadzu (version 5.97, Shimadzu, Duisburg, Germany).

### 3.5. Validation

Method validation was performed according to FDA guidance for bioanalytical methods dated May 2018 [17]. The analytical parameters calibration curve, selectivity, sensitivity, accuracy, precision, recovery, matrix effect, and stability were investigated. The parameters were validated by evaluating the ratios of the product mass transition signals of the respective analyte peak and the peak of the internal standard. Calibration curves were generated by a simple linear regression which included a 1/x weighting using the least squares method.

Calibration curves were generated using analyte concentrations from 0.67 nM to 333 nM for KD87, KD85, and I3M precipitated from plasma samples (0.67, 1.33, 2.0, 3.3, 6.7, 10.0, 13.3, 16.7, 20.0, 26.7, 33.3, 66.7, 116, 167, and 333 nM). For all analytes extracted from plasma, the calibration range covered fifteen levels from 1 to 500 nM (1, 2, 3, 5, 10, 15, 20, 25, 30, 40, 50, 100, 175, 250, and 500 nM). In contrast, the calibration curve generated for DMEM extraction was reduced to eleven levels, and ranged from 1 to 50 nM (1, 2, 3, 5, 10, 15, 20, 25, 30, 40, and 50 nM). In accordance with FDA guidance [17], 75% of these concentrations and at least 6 non-zero calibrators were used to evaluate the calibration curves. Indirubin was added to each sample as an internal standard as previously described. 

Four control sample concentrations were set for each analyte for all three processing methods. The lower limit of quantitation was 0.67 nM (protein precipitation) and 1 nM (plasma and DMEM extraction), the low concentration was 2 nM (protein precipitation) and 3 nM (plasma and DMEM extraction), the medium concentration was 26.7 nM (protein precipitation), 40 nM (plasma extraction), and 15 nM (DMEM extraction), and the high concentration was 167 nM (protein precipitation), 250 nM (plasma extraction), and 40 nM (DMEM extraction).

For the selectivity of plasma extraction and protein precipitation, six different human plasmas were analyzed as required by FDA guidance [17]. For selectivity of DMEM extraction, two different high glucose media were analyzed.

Sensitivity was determined by evaluating LLOQ with a S/N of at least 5 by applying the root mean square method using LabSolutions LCMS analysis software (version 5.97) without smoothing.

Possible carryover was determined by injecting 10 µL of the highest calibration concentration followed by a cleaning run with acetonitrile/water (50/50). If no carryover was observed, the sample was injected twice in succession before starting another cleaning run.

For accuracy, represented as relative error (RE; [(measured concentration − nominal concentration)/nominal concentration] × 100%), a deviation from nominal concentration of ±20% for LLOQ and ±15% for other concentrations used was allowed. As a measure of precision, the coefficient of variation (CV; standard deviation/mean of measured concentration × 100%) was set at ±20% for LLOQ and ±15% for other concentrations used, as required by FDA guidance [17]. To determine accuracy and precision, a complete calibration curve was constructed with blank, zero calibrator, and control samples consisting of LLOQ, low, medium, and high concentrations and prepared with *n* = 5–6. At least five control samples were included in the evaluation of intra-day accuracy and precision. Four measurements of intra-day accuracy and precision were made and used to determine the inter-day accuracy and precision of the methods with *n* = 20–24.

The recovery (calculated as: (sample signal/postspiked signal) × 100%) of each analyte from plasma and DMEM was determined for all three processing methods using at least three independent samples at low, medium, and high concentrations. Here, the matrix was spiked with the analyte concentrations prior to the start of the sampling procedure and compared to the areas obtained from a blank matrix spiked with the analyte concentration after the processing was completed. In protein precipitation, all analytes were added to the matrix during the precipitation reaction, whereas in liquid-liquid extraction, the post-spiked samples were prepared by adding the analytes after evaporation of the blank. In each case, the internal standard was added prior to analysis. For recovery of the internal standard, the absolute values of the areas were compared. For all investigations, *n* = 3–5 replicates were prepared.

An additional requirement of an LC-MS/MS method is the determination of the matrix effect (calculated as: [1 − (postspiked signal/stock solution signal)] × 100%) to gain insight into possible ion suppression or ion amplification by matrix components leading to a change in signal response. To determine the matrix effect, each analyte and both extraction methods were analyzed at low, medium, and high concentrations. For this purpose, the matrix was extracted and spiked with either the analytes or the internal standard. These samples were compared with the stock solution standards of the compounds and the obtained analyte to internal standard ratios were compared. For all investigations, *n* = 3–5 replicates were prepared.

To assess stability, low and high concentrations of all analytes were exposed to different storage conditions. To determine long-term stability, samples were stored in either plasma or DMEM at −80 °C for 90 and 91 days, respectively. To determine short-term stability, samples were stored for 24 h on the bench at room temperature, in the autosampler, at 4 °C, and at −20 °C. In addition, freeze-thaw cycles were performed for the standard solutions according to FDA guidance [17]. At least three freshly prepared samples per concentration level were prepared for comparison. With the exception of the stability test in the autosampler, the internal standard was always added to all samples before sample processing was started. However, to avoid concentration fluctuations due to possible degradation of the analytes, stock solutions were prepared twice a week and stored at −20 °C. Each storage condition was examined in the dark. For all conditions studied, *n* = 3–6 replicates were prepared.

### 3.6. Animal Experiment and Mouse Plasma Collection

NOD.Cg-*Prkdc^scid^ Il2rg^tm1Wjl^*/SzJ (NSG) mice were initially purchased from Jackson Laboratory. These mice are extremely immunodeficient due to two mutations, namely severe combined immunodeficiency (*scid*) and a complete null allele of the IL2 receptor common gamma chain (*IL2rg^null^*). The health status of the animals was routinely checked according to FELASA guidelines. NSG mice were kept at a 12 h light/dark cycle and an ambient temperature of 21 ± 2 °C with 60 ± 20% relative humidity. Water and standard laboratory feed were provided ad libitum. Mice were bred under specific germ-free conditions in individually ventilated cages and with environmental enrichment. 

For experiments, four 13-week-old male NSG mice with a body weight between 20 and 30 g were used. KD87 was administered via intraperitoneal injection at a dose of 20 mg/kg body weight. For this purpose, KD87 was dissolved in ethanol and Cremophore EL (1:1) to a concentration of 6 mg/mL, and diluted further in sterile saline solution (NaCl 0.9%, B. Braun Melsungen AG, Melsungen, Germany) to a final concentration of 2 mg/mL. Per mouse, a total volume of 10 mL/kg of body weight was administered. Blood samples were collected from each mouse 30 or 60 min after KD87 injection. For retro-orbital blood collection, mice were anesthetized with 1.5–2.5% isoflurane (CP-pharma, Burgdorf, Germany) in oxygen. Immediately after the second blood sampling, the animals were sacrificed by cervical dislocation. 

Whole blood was treated with ethylenediaminetetraacetic acid as anticoagulant and centrifuged to separate plasma and red blood cells. Samples were stored at −80 °C until sample preparation was performed. For both precipitation and plasma extraction, each sample was diluted 1:5 with human plasma, which was also considered in the subsequent analysis. For each plasma sample obtained, two aliquots were processed for separate sample preparation and subsequent measurement. 

## 4. Conclusions

In summary, the developed and validated methods provide useful and reliable assay tools to gain new insights into the pharmacokinetics of antitumor acting thia-analogous indirubin *N*-glycosides in vivo and in vitro. Furthermore, the methods have the potential to be modified to allow quantification of additional glycosylated indirubin analogs and derivatives.

Although, based on the validations performed, the quantification of the investigated compounds in cell culture experiments is limited to the extraction procedure, both extraction and precipitation can be used for plasma analyses in the context of in vivo studies. In this context, precipitation appears particularly suitable when analysis must be rapid and storage is limited to temperatures below 0 °C, whereas extraction provides more accurate and precise results. Since validation was performed in human plasma, applicability of the presented method in future clinical studies is possible.

## Figures and Tables

**Table 1:** Applied mass spectrometer and source parameters. *m*/*z* quantifiers are marked with *, retention time (t_R_), mass-to-charge-ratio (*m*/*z*), milliseconds (ms), and volt (V).

Parameters for LC		Parameters for MS				
Compound	t_R_ (min)	ESI	Precursor *m*/*z*	Product *m*/*z*	Dwell Time (ms)	Collision Energy (V)
KD87	4.1	+	442.0	280.0 * 292.0	72.0	−16.0
KD85	4.8	+	442.0	280.0 * 292.0	72.0	−16.0
I3M	5.6	+	278.0	261.1 * 205.0	58.0	−16.0
I3M	5.6	-	276.1	246.2 * 157.0	181.0	17.0
Indirubin	6.7	+	263.2	219.1 * 234.8	123.0	−24.0

**Table 2:** Inter- and intra-day accuracy, expressed as relative error (RE) ± standard deviation (SD), and precision, determined as coefficient of variation (CV), of different samples each from four days of KD87, KD85, and I3M at LLOQ level (0.67 nM), low (2 nM), medium (26.7 nM), and high (167 nM) concentrations after plasma precipitation. The measured concentrations are given as mean values ± SD, *n* = 5–6 for intra-day, *n* = 20–24 for inter-day.

	KD87					KD85					I3M				
Added Concentration (nM)	Intra-Day			Inter-Day		Intra-Day			Inter-Day		Intra-Day			Inter-Day	
	Measured Concentration (nM)	RE (%)	CV (%)	RE (%)	CV (%)	Measured Concentration (nM)	RE (%)	CV (%)	RE (%)	CV (%)	Measured Concentration (nM)	RE (%)	CV (%)	RE (%)	CV (%)
0.67	0.66 ± 0.02	−0.95 ± 2.9	2.91	−0.95 ± 6.5	6.53	0.67 ± 0.03	0.93 ± 4.7	4.69	0.98 ± 5.7	5.62	0.65 ± 0.01	−2.48 ± 2.2	2.26	−2.49 ± 7.0	7.21
2.00	2.01 ± 0.08	0.35 ± 4.1	4.07	0.35 ± 4.7	4.72	1.98 ± 0.07	−1.03 ± 3.5	3.51	−1.03 ± 4.2	4.25	2.04 ± 0.09	1.99 ± 4.6	4.52	1.99 ± 5.4	5.27
26.7	26.09 ± 0.39	−2.18 ± 1.5	1.49	−2.19 ± 2.9	2.91	26.02 ± 0.36	−2.43 ± 1.4	1.39	−2.43 ± 2.7	2.79	26.97 ± 0.66	1.15 ± 2.5	2.46	1.06 ± 4.1	4.05
167	165.8 ± 1.13	−0.51 ± 0.68	0.68	−0.51 ± 1.9	1.90	166.4 ± 0.63	−0.16 ± 0.38	0.38	−0.15 ± 1.3	1.33	165.5 ± 5.63	−0.68 ± 3.4	3.40	−0.68 ± 4.0	4.01

**Table 3:** Inter- and intra-day accuracy, expressed as relative error (RE) ± standard deviation (SD) and precision, determined as coefficient of variation (CV), of different samples each from four days of KD87, KD85, and I3M at LLOQ level (1 nM), low (3 nM), medium (40 nM), and high (250 nM) concentrations after extraction from plasma. The measured concentrations are given as mean values ± SD, *n* = 5–6 for intra-day, *n* = 20–24 for inter-day.

	KD87					KD85					I3M				
Added Concentration (nM)	Intra-Day			Inter-Day		Intra-Day			Inter-Day		Intra-Day			Inter-Day	
	Measured Concentration (nM)	RE (%)	CV (%)	RE (%)	CV (%)	Measured Concentration (nM)	RE (%)	CV (%)	RE (%)	CV (%)	Measured Concentration (nM)	RE (%)	CV (%)	RE (%)	CV (%)
1.0	0.99 ± 0.03	−0.67 ± 2.7	2.67	−0.62 ± 5.0	4.99	1.00 ± 0.006	−0.04 ± 0.6	0.59	−0.02 ± 3.1	3.12	1.02 ± 0.05	2.24 ± 5.1	5.01	2.24 ± 7.1	6.89
3.0	2.97 ± 0.09	−1.14 ± 3.1	3.09	−1.14 ± 4.1	4.14	2.93 ± 0.05	−2.47 ± 1.6	1.61	−2.57 ± 4.1	4.20	2.99 ± 0.13	−0.42 ± 4.2	4.21	−0.66 ± 5.8	5.82
40	39.4 ± 1.14	−1.59 ± 2.8	2.88	−1.59 ± 3.6	3.67	39.2 ± 0.91	−1.97 ± 2.3	2.33	−1.99 ± 2.8	2.84	39.8 ± 0.61	−0.59 ± 1.5	1.53	−0.71 ± 3.1	3.11
250	241.7 ± 7.29	−3.32 ± 2.9	3.01	−3.32 ± 4.2	4.34	243.4 ± 7.03	−2.66 ± 2.8	2.89	−2.67 ± 3.6	3.74	242.2 ± 2.79	−3.14 ± 1.1	1.15	−3.07 ± 2.6	2.71

**Table 4:** Inter- and intra-day accuracy, expressed as relative error (RE) ± standard deviation (SD) and precision, determined as coefficient of variation (CV), of different samples each from three days of KD87, KD85, and I3M at LLOQ level (1 nM), low (3 nM), medium (15 nM), and high (40 nM) concentrations after extraction from DMEM. The measured concentrations are given as mean values ± SD, *n* = 5–6 for intra-day, *n* = 20–24 for inter-day.

	KD87					KD85					I3M				
Added Concentration (nM)	Intra-Day			Inter-Day		Intra-Day			Inter-Day		Intra-Day			Inter-Day	
	Measured Concentration (nM)	RE (%)	CV (%)	RE (%)	CV (%)	Measured Concentration (nM)	RE (%)	CV (%)	RE (%)	CV (%)	Measured Concentration (nM)	RE (%)	CV (%)	RE (%)	CV (%)
1.0	1.03 ± 0.02	2.71 ± 1.8	1.75	2.71 ± 3.5	3.37	0.99 ± 0.04	−1.13 ± 3.9	3.96	−1.00 ± 6.5	6.55	0.99 ± 0.04	−0.80 ± 3.7	3.73	−0.64 ± 4.4	4.45
3.0	3.03 ± 0.11	1.00 ± 3.6	3.60	1.00 ± 4.5	4.50	3.0 ± 0.08	0.16 ± 2.5	2.51	0.16 ± 5.3	5.32	3.07 ± 0.05	2.43 ± 1.6	1.56	2.35 ± 2.9	2.85
15	15.07 ± 0.26	0.49 ± 1.7	1.70	0.59 ± 2.9	2.88	14.49 ± 0.45	−3.38 ± 3.0	3.13	−3.27 ± 3.3	3.43	15.18 ± 0.07	1.18 ± 0.5	0.47	1.18 ± 2.1	2.12
40	39.86 ± 0.67	−0.35 ± 1.7	1.69	−0.42 ± 2.6	2.63	39.15 ± 1.06	−2.13 ± 2.7	2.72	−2.19 ± 3.5	3.60	40.13 ± 0.25	0.31 ± 0.6	0.61	0.34 ± 0.6	1.43

**Table 5:** Recovery of the analytes KD87, KD85, I3M, and the internal standard (IS) after plasma precipitation, plasma extraction, or DMEM extraction. Values are mean percentages ± standard deviation (SD) of *n =* 3–5.

Plasma Precipitation						Plasma Extraction						DMEM Extraction					
c (nM)	KD87	KD85	I3M	IS		c (nM)	KD87	KD85	I3M	IS		c (nM)	KD87	KD85	I3M	IS 5 µL	IS 2 µL
50				97.0 ± 2.1		50				64.3 ± 1.3		50				83.4 ± 5.1	85.4 ± 4.2
2.0	92.77 ± 6.1	90.20 ± 7.7	107.09 ± 4.5			3.0	87.94 ± 5.4	92.86 ± 4.5	105.3 ± 2.6			3.0	64.36 ± 1.5	59.97 ± 1.8	71.67 ± 0.5		
26.7	86.64 ± 2.7	87.67 ± 3.6	92.34 ± 5.0			40	87.39 ± 4.1	88.75 ± 3.7	92.17 ± 2.8			15	61.90 ± 0.9	62.95 ± 3.0	71.25 ± 2.2		
167	89.67 ± 1.5	89.27 ± 1.5	96.74 ± 10.8			250	87.43 ± 1.8	86.94 ± 0.4	92.08 ± 6.9			40	70.46 ± 2.3	64.75 ± 2.3	69.95 ± 1.4		

**Table 6:** Matrix effect for analytes KD87, KD85, I3M, and internal standard (IS) after plasma extraction or DMEM extraction. Values are mean percentages ± standard deviation (SD) of *n =* 3–5.

Plasma Extraction						DMEM Extraction					
c (nM)	KD87	KD85	I3M	IS		c (nM)	KD87	KD85	I3M	IS 5 µL	IS 2 µL
50				2.68 ± 3.2		50				2.13 ± 2.9	1.96 ± 2.2
3.0	0.94 ± 1.4	0.61 ± 1.5	−0.55 ± 3.6			3.0	0.68 ± 4.7	−0.61 ± 2.4	1.46 ± 5.5		
40	−0.08 ± 3.3	1.01 ± 2.1	2.57 ± 1.2			15	1.20 ± 1.8	5.97 ± 1.1	2.93 ± 3.6		
250	3.15 ± 1.2	1.31 ± 2.9	−1.12 ± 4.1			40	3.95 ± 3.2	1.74 ± 1.5	1.83 ± 4.0		

**Table 7:** Stability of KD87, KD85, and I3M analytes after plasma precipitation, plasma extraction, or DMEM extraction under various storage conditions, i.e., benchtop (BT), 4 °C, −20 °C, autosampler (AS), freeze-thaw (FT), and long-term freezing (90 or 91 days at −80 °C). The duration of storage was 24 h unless stated otherwise. Values are mean percentages ± standard deviation (SD) of *n =* 3–6.

	Plasma Precipitation								Plasma Extraction								DMEM Extraction						
	c (nM)	BT	4 °C	−20 °C	AS	FT	−80 °C 91 d		c (nM)	BT	4 °C	−20 °C	AS	FT	−80 °C 90 d		c (nM)	BT	4 °C	−20 °C	AS	FT	−80 °C 91 d
KD87	2.0	64.3 ± 5.6	81.4 ± 3.1	94.6 ± 2.8	105.7 ± 6.7	98.8 ± 5.6	96.7 ± 8.6	KD87	3.0	64.4 ± 4.6	79.3 ± 2.4	101.4 ± 3.4	80.8 ± 3.0	98.3 ± 5.4	100.1 ± 8.4	KD87	3.0	104.9 ± 4.0	81.4 ± 3.3	87.7 ± 3.2	101.2 ± 7.4	98.3 ± 5.4	97.8 ± 6.0
	167	75.0 ± 3.5	84.5 ± 2.0	92.6 ± 2.6	99.9 ± 4.8	97.9 ± 4.1	106.0 ± 1.4		250	77.8 ± 2.7	94.1 ± 5.6	100.1 ± 0.7	73.7 ± 3.3	99.7 ± 2.6	100.9 ± 3.6		40	98.3 ± 1.1	84.5 ± 2.1	84.2 ± 2.6	81.7 ± 3.7	101.9 ± 5.4	92.3 ± 7.8
KD85	2.0	57.5 ± 4.1	81.3 ± 3.4	90.1 ± 5.7	97.6 ± 8.0	107.9 ± 4.9	105.1 ± 12.4	KD85	3.0	64.4 ± 3.6	85.5 ± 2.2	86.4 ± 3.5	71.8 ± 2.7	99.4 ± 4.0	102.5 ± 5.2	KD85	3.0	115.3 ± 1.8	98.3 ± 2.2	86.8 ± 1.8	75.6 ± 5.8	99.4 ± 4.0	93.2 ± 6.9
	167	70.3 ± 3.2	85.5 ± 1.9	96.3 ± 2.3	99.6 ± 4.1	99.5 ± 3.8	108.2 ± 3.7		250	76.0 ± 2.6	94.3 ± 5.7	101.8 ± 3.2	73.1 ± 2.7	99.1 ± 2.0	101.7 ± 1.1		40	100.5 ± 1.9	80.5 ± 1.8	81.9 ± 3.9	71.6 ± 1.9	99.9 ± 3.3	92.7 ± 5.7
I3M	2.0	100.1 ± 9.0	89.7 ± 1.5	95.2 ± 3.7	124.3 ± 9.5	104.1 ± 5.5	101.0 ± 9.0	I3M	3.0	102.2 ± 1.8	95.9 ± 8.7	93.8 ± 1.9	68.0 ± 1.5	94.9 ± 4.0	96.2 ± 7.2	I3M	3.0	66.8 ± 2.7	113.8 ± 6.6	92.0 ± 1.1	65.0 ± 1.3	100.0 ± 5.7	90.6 ± 4.5
	167	98.6 ± 4.3	91.5 ± 3.3	93.7 ± 7.7	112.0 ± 6.8	99.7 ± 7.4	103.0 ± 3.6		250	96.3 ± 7.3	94.6 ± 2.2	96.5 ± 1.6	58.5 ± 1.3	99.7 ± 1.6	100.6 ± 3.0		40	59.4 ± 2.4	100.4 ± 1.3	93.1 ± 2.5	63.6 ± 0.3	99.5 ± 3.2	99.0 ± 2.7

## Data Availability

The data presented in this study are available on reasonable request from the first author.

## Sample Availability

Not available.
